# Examining the Economic Perspective of Treatable Mortality: The Role of Health Care Financing and the Importance for Economic Prosperity

**DOI:** 10.3389/fpubh.2021.780390

**Published:** 2021-12-13

**Authors:** Viera Ivankova, Beata Gavurova, Samer Khouri, Gabriel Szabo

**Affiliations:** Institute of Earth Resources, Faculty of Mining, Ecology, Process Control and Geotechnologies, Technical University of Košice, Košice, Slovakia

**Keywords:** economic productivity, health systems, expenditure, treatable mortality, gross domestic product, gender classification, OECD countries, working age population

## Abstract

Health is an essential element of economic life and is therefore considered a source of comparative economic development of countries. The aim of the study was to examine the associations between health care financing, specific treatable mortality of males and females of working age, and economic prosperity, taking into account to the classification of health systems applied in the countries of the Organization for Economic Co-operation and Development (OECD). An insurance-based health system and a tax-based health system were identified in these countries, and data were collected for the period 1994–2016. Descriptive analysis, panel regression analysis and cluster analysis were used to achieve the aim. The analytical process included economic indicators [health expenditure, gross domestic product (GDP)] and health indicators (treatable mortality from circulatory system diseases and endocrine, nutritional and metabolic diseases). The results revealed significant negative associations of health care financing with treatable mortality from circulatory system diseases and endocrine, nutritional, and metabolic diseases in both health systems and both gender categories. There were also negative associations between treatable mortality in both diagnosis groups and economic prosperity. These results have shown that health care financing is linked to economic prosperity also through health variability in the working age population. In terms of assessing economic and health outcomes, less positive and more positive countries were identified using cluster analysis. Countries such as Latvia with a tax-based health system and Hungary, Lithuania, Estonia with an insurance-based health system were characterized by great potential for improvements. Although reducing treatable mortality is a great motivation for public health leaders to increase health care financing, the importance for economic prosperity may be a more compelling argument. Effective interventions should be considered in the light of their regional, social and economic contexts.

## Introduction

The current global situation underlines the need to focus on public health in order to eliminate unnecessary deaths and ensure economic growth. The economic impact of health is well-known, and therefore measures leading to better population health contribute to the creation of richer economies (1). In this context, health care can be considered as one of the important factors in the economic development of countries (2). It is also true that the provision of health care depends to a large extent on its financing, and therefore if countries strive to have a healthy productive population, they must also focus on the financing of health care (3). These facts suggest that health care financing, health and economic prosperity are important and interconnected elements of any country's life and should be at the center of both professional and political attention.

In line with the above-mentioned facts, the main aim of the study was to examine the associations between health care financing, specific treatable mortality of males and females of working age, and economic prosperity, taking into account to the classification of health systems applied in OECD countries. The main idea of the research was based on the assumption that the way and level of health care financing could be reflected in specific health outcomes, which can also affect the economic prosperity of OECD countries. If health systems are well-designed, they should achieve comparable results. The importance of the issue is unquestionable due to the ongoing pandemic, which has caused health and economic losses around the world, but the need for attention has also been before it. The continuing need for research into treatable mortality is underlined by the importance of this indicator, which is poorly investigated in the economic dimension. The results of the presented study are important for understanding the extent to which health and economic elements are interconnected, in a diverse classification according to the applied health system and gender characteristics of the working age population. Knowledge of these results makes it possible to design optimal forms of health policies at the national and international levels in order to eliminate health inequalities not only within individual countries but also between countries. Along with health improvements, leaders can expect economic benefits.

## Theoretical Background

It is well-known that health is a value in itself, it is also a prerequisite for economic prosperity. Population health is linked to economic performance in terms of productivity, labor supply, human capital, and public expenditure. In this context, individuals in good health represent the driving force of the economy, but individuals in poor health spend public resources and represent a potential burden in times of disease and death. Therefore, the issue of health and economic life requires an increased attention of researchers, professionals and policy makers in order to improve the health of the population. One of the many ways to improve it is adequate financing. Health expenditure can ensure the good health of individuals which can increase the volume and productivity of their work and bring benefits to the economy (4). In this context, the findings of Murphy and Topel (5) need to be emphasized, which have clearly shown that improving the health of the population in terms of increasing life expectancy and reducing mortality translates into increased economic value of health capital, bringing significant economic gains. Based on all these findings, it is true that health is essential for economic prosperity and represents economic development (6).

The relationship between health care financing and public health has received considerable attention in recent decades, and the authors of several international studies support the idea that health outcomes are influenced, among other factors, by the level of health care financing (7–9). Novignon et al. (10) revealed that higher health expenditure has a significant effect on population health, especially in terms of higher life expectancy and lower mortality. On this basis, it can be stated that an adequate level of financing in health care plays an important role in improving health outcomes in a country (3, 11, 12). On the other hand, there are findings that indicate that health expenditure is not the only determinant of health (13). This underlines the ambiguity and complexity of the relationship between health expenditure and health outcomes, which creates problems in international comparison (14).

Although health care financing varies from country to country, it should be based on pillars such as solidarity and equity, in order to provide accessible and quality health care. That is the core of modern health systems applied in countries based on tax or insurance principles. At the macroeconomic level, the overall effectiveness of health systems is assessed in terms of improving health, while health outcomes are often confronted with health expenditure (15). Van der Zee and Kroneman (16) compared the effectiveness of an insurance-based health system and a tax-based health system with a focus on health care financing, health outcomes and patient satisfaction. In their results, the authors revealed that the system based on health insurance acquired a more favorable assessment in terms of overall mortality, life expectancy, as well as patient satisfaction. On this basis, differences in health outcomes can be expected between countries with different health systems, and this aspect should be taken into account when examining the link between health and economic outcomes.

The issue under investigation provides many health indicators as appropriate measures. One of the most commonly used indicators of population health is mortality, and the results of many studies support the claim that reducing population mortality improves aspects of economic life in countries such as economic development and prosperity (17–19). On the other hand, treatable mortality is considered a key indicator demonstrating the extent of the contribution of health care to the health status of the population (20, 21). Treatable mortality is part of the concept of avoidable mortality that is based on the idea that, with current medical knowledge and technology, certain deaths could be averted, in particular through effective interventions in health systems (prevention and treatment). This means that overall mortality would not reach such a level if effective prevention and adequate health care were provided (22). It follows that treatable mortality, as part of avoidable mortality, is linked to the financing of health care. This fact was proved by Heijink et al. (23), who confirmed a statistically significant negative association between avoidable mortality and health expenditure using fixed-estimate regression models. The authors emphasized that most countries with above-average growth in health expenditure reported above-average decline in avoidable mortality. Reduction of treatable mortality is desirable, as treatable mortality represents an economic burden. According to Alkire et al. (24), the unfoundedness of these deaths brings economic losses in the form of a decline in countries' GDP.

The above-mentioned facts underline the importance of examining the associations between treatable mortality and economic outcomes. As could be seen, the link between health and economic life has been often examined in one dimension, while this study applies multilevel research, first the association between health care financing and treatable mortality, and then the association between treatable mortality and economic prosperity. Health policy makers should know how health care financing can be reflected in economic prosperity through population health represented by treatable mortality. The novelty of the presented study also lies in the international comparison of OECD countries classified according to their applied health system. In addition to the classification according to health systems, the study respects gender differentiation in treatable mortality from circulatory system diseases and endocrine, nutritional and metabolic diseases. This allows a deeper insight into the issue.

## Methodology

### Research Aim and Questions

The main aim of the study was to examine the associations between health care financing, specific treatable mortality of males and females of working age, and economic prosperity, taking into account to the classification of health systems applied in OECD countries. Based on this aim, the following research questions were formulated:

RQ1: Are there significant associations between health care financing and selected diseases as a cause of treatable mortality for males and females of working age in OECD countries that apply a tax-based health system?

RQ2: Are there significant associations between health care financing and selected diseases as a cause of treatable mortality for males and females of working age in OECD countries that apply an insurance-based health system?

RQ3: Are there significant associations between selected diseases as a cause of treatable for males and females of working age and economic prosperity in OECD countries that apply a tax-based health system?

RQ4: Are there significant associations between selected diseases as a cause of treatable mortality for males and females of working age and economic prosperity in OECD countries that apply an insurance-based health system?

### Research Data

For the purposes of this research, data were collected from available OECD (25, 26) and World Health Organization (WHO) (27) databases. These data were collected for all available years, with the oldest data from 1994 and the most recent data from 2016. In this sense, it is possible to define the analyzed period from 1994 to 2016. The variables analyzed in this research can be divided into two groups according to their nature:

- Economic variables – health care financing, economic prosperity of countries represented by their GDP,
- Health variables – treatable mortality of males and females with a closer look at diseases of the circulatory system and endocrine, nutritional and metabolic diseases.

The financing of health care represented total health expenditure as a percentage of countries' GDP. Health expenditure expressed the final consumption of health products and services, including personal health care (medical care, rehabilitation care, long-term care, ancillary services, and medical goods/materials) and collective services (prevention, public health services, health administration) (26).

Economic prosperity covered the GDP of countries, which is a standard measure of the added value generated in a country. GDP is considered to be one of the most important outcome indicators that capture countries' economic activity. This indicator was based on GDP expressed in dollars and converted per capita (in current PPP prices) (25).

Health variables included mortality from treatable diseases in males and females, specifically circulatory system diseases and endocrine, nutritional and metabolic diseases. These diagnosis groups were selected on the basis that they mainly include non-communicable diseases, which tend to be long-lasting. Treatable mortality is mortality from causes that can be avoided in particular by early and effective interventions in health care, including secondary prevention and treatment (after the onset of diseases to reduce mortality from treatable causes) (28). The calculation of the treatable mortality rate was based on the sum of standardized mortality for specific causes and data were collected from the WHO database (27). As indicated above, the research covered two diagnosis groups of treatable diseases with regard to the OECD and Eurostat list (28). Each group consisted of specific treatable diseases identified by the 10th version of the International Classification of Diseases (ICD-10):

- Circulatory system diseases: I01–I26, I60–I71, I73, I80, I82,
- Endocrine, nutritional and metabolic diseases: E00–E14, E24–E25, E27, E74.

Numbers of deaths from these treatable causes were collected across countries for all available years for which countries reported a value (including zero). Data were available for various age categories separately for males and females. The data collected on treatable deaths were recalculated per 100,000 inhabitants of a given country in a specific gender and age category (i.e., per 100,000 males aged 25–64 years in a given country and per 100,000 females aged 25–64 years in a given country). This step was preceded by the collection of population data in this specific gender and age category for each country and each analyzed year, when individual treatable deaths were reported. Population data were provided from the United Nations database as part of their report “World Urbanization Prospects” (29).

From a macroeconomic point of view, productivity is the connecting element of all analyzed variables and the associations between them. The selected indicators present the level of health care financing in countries as a percentage of their productivity (GDP), the treatable mortality of the working age population and the level of economic productivity (GDP) as such. The age category was selected on the basis of several studies examining the age and productivity of the population (30–32), but it is also generally assumed that people enter the labor market from the moment of graduation and leave it at retirement age. At the same time, the age of 25 is a period of life in which individuals are likely to have found a targeted job or have already established themselves in the labor market, even if they have not graduated from university.

### Research Subjects

The research sample consisted of countries that have developed a health system for the provision of primary and specialized (secondary and tertiary) health care. Thus, 37 OECD countries were included in the analytical process. The classification of OECD countries in terms of applied health system was performed on the basis of data from surveys of health systems characteristics in OECD countries (33, 34), on the basis of data from country health profiles (35, 36), as well as on the basis of data provided on the websites of the Ministry of Health of each country included in the research. It should also be noted that Latvia replaced the principle of health care financing from an insurance-based system to a tax-based system in 2011 and therefore only the most recent data were taken into account. The classification of countries was as follows:

- Tax-based health system: Australia (AU), Canada (CA), Denmark (DK), Finland (FI), Iceland (IS), Ireland (IE), Italy (IT), Latvia (LV), Norway (NO), New Zealand (NZ), Portugal (PT), Spain (ES), Sweden (SE), the United Kingdom (GB);
- Insurance-based health system: Austria (AT), Belgium (BE), Chile (CL), Colombia (CO), the Czech Republic (CZ), Estonia (EE), France (FR), Germany (DE), Greece (GR), Hungary (HU), Israel (IL), Japan (JP), Korea (KR), Lithuania (LT), Luxembourg (LU), Mexico (MX), the Netherlands (NL), Poland (PL), Slovakia (SK), Slovenia (SI), Switzerland (CH), Turkey (TR), the United States (US).

Based on this classification of countries, it can be seen that a tax-based health system was applied by a total of 14 countries and an insurance-based health system was applied by 23 countries.

### Statistical Analysis

The assessment of the collected data was performed in the first step of the statistical processing through a descriptive analysis, which provided a closer look at the economic and health outcomes in OECD countries. In this context, central tendency measures (arithmetic mean, median), variability measure (standard deviation), quartiles, minimum and maximum, as well as position measures (skewness and kurtosis) were used in this analysis. It was the position measures in most of the cases that indicated a possible disruption of the normal distribution of data. Hair et al. (37) declare that values of skewness and kurtosis in the range of −1 to +1 are indicators of normal distribution, but most cases in this study exceeded this range. The structure of the data indicated a disruption of the normal distribution as well as the presence of outliers.

Regression analysis was used to evaluate the significance of the associations between health care financing, treatable mortality in selected diagnosis groups, and the economic prosperity of countries. Robust methods were used to estimate the coefficients, specifically models with random or fixed effects in one-way (individual) or two-ways variants. The regression analysis was preceded by panel diagnostics to select appropriate regression models using the F test for the presence of individual effects (or time effects) and the Hausman test. The one-way variant was supported if a statistically significant effect was demonstrated only within countries or only within years, then the effect was taken into account in the corresponding model. As pointed out in the following section, if there was only one significant effect in the data structure, it was the country structure, not the time structure. Thus, the one-way variant of the models always took into account the effects identified in the country structure. On the contrary, the two-ways variant was supported if a statistically significant effect was demonstrated both within countries and within years (time effect), and these effects were taken into account in the relevant models. Subsequent decision-making processes led to a preference for a model with random or fixed effects, and Hausman's test statistics helped with this choice. If the result of the Hausman test confirmed the significance at the level α < 0.05, the preference was in favor of choosing a model with fixed effects (the null hypothesis was rejected). Otherwise (α > 0.05), the choice was focused on a model with random effects (the null hypothesis was not rejected).

The HC3 estimator was used to estimate the regression coefficients, with respect to the data showing outliers and heteroscedasticity. The Arellano estimation method (38) for fixed effect models and the White 2 estimation method for random effects models were used to assess the significance of the coefficients. Finally, the associations were examined in four variants of regression models with a preference for one of them (based on the above panel assumptions): “One-way (individual) effect Fixed effect model” (Arellano), “One-way (individual) effect Random effect model” (White 2), “Two-ways effects Within (fixed) effect model” (Arellano), “Two-ways effects Random effect model” (White 2).

In the last third step of the statistical processing, a cluster analysis of the links between economic outcomes and treatable mortality outcomes was performed in the specification of gender and health systems. The number of clusters was supported by the result of the silhouette method (39), while the Partitioning Around Medoids (PAM) method based on the Manhattan distance was used to determine the clusters (40). The output of this analysis offered a classification of countries.

The analytical processing was performed in the programming language R v 4.1.1 (RStudio, Inc., Boston, MA, USA).

## Results

### Descriptive Statistic—Univariate View

This section focuses on the statistical description of variables to provide a closer look at economic and health outcomes across OECD countries.

Based on the results in Table 1, the economic variables can be interpreted as follows. In countries with a tax-based health system, health care financing averaged 8.68% of GDP during the analyzed period, with a minimum of 5.4% (Latvia in 2013) and a maximum of 10.98% (Sweden in 2014). Health care financing in countries with an insurance-based health system averaged 7.82% of GDP, with a minimum of 3.35% of GDP (Korea in 1995) and a maximum of 16.71% of GDP (the United States in 2015). Thus, it is clear that countries with an insurance-based health system financed health care at a lower level than countries with a tax-based health system. Countries with an insurance-based health system also showed lower economic prosperity, as evidenced by a mean of 28,591.4 USD per capita compared to a mean of 35,565.1 USD per capita in countries with a tax-based health system.

**Table 1:** Descriptive statistic of economic and health variables classified by health systems and gender (1994–2016).

	**N**	**Miss**	**Mean**	**Median**	**St Dev**	**Skew**	**Kurt**	**Min**	**Max**	**1st Q**	**3rd Q**
**Tax-based health system**											
HF	216	0	8.68	8.69	1.07	−0.25	0.55	5.4	10.98	8.02	9.32
GDP	216	0	35,565.1	34,198.6	9,402.5	0.8	0.78	19,887.8	66,956.3	28,704.9	41,523.5
CRC–M	216	0	74.44	65.73	37.99	3.38	14.77	27.25	289.23	53.53	81.91
CRC–F	216	0	25.32	22.9	12.04	3.21	14.87	3.67	99.1	18.17	29.40
END–M	216	0	6.59	6.3	3.02	0.44	−0.33	<0.01	14.79	4.08	8.84
END–F	216	0	3.38	3.22	1.65	0.46	−0.20	<0.01	7.66	2.24	4.35
**Insurance-based health system**											
HF	389	17	7.82	7.15	2.45	1.16	1.76	3.35	16.71	6.08	9.55
GDP	406	0	28,591.4	26,585.6	16,312.2	1.51	3.63	6,554.6	103,788	16,451.5	35,896.2
CRC–M	406	0	110.53	71.74	79.8	1.37	0.78	29.26	386.57	60.34	149.84
CRC–F	406	0	41.35	33.51	26.97	1.3	1.17	7.69	141.41	21.77	50.66
END–M	406	0	10.61	7.77	11.49	3.96	15.84	1.55	71.31	5.72	10.8
END–F	406	0	7.12	4.33	10.33	3.96	15.18	0.66	56.4	2.77	7.44

*HF, Health care financing in % of GDP; GDP, Gross domestic product per capita; F, Females; M, Males, CRC, Treatable mortality from circulatory system diseases per 100,000 males/females aged 25–64 years; END, Treatable mortality from endocrine, nutritional and metabolic diseases per 100,000 males/females aged 25–64 years; N, number of observations, Miss, Missing values; St Dev, Standard deviation; Skew, Skewness; Kurt, Kurtosis; Min, Minimum; Max, Maximum; 1st Q, First quartile; 3rd Q, Third quartile*.

With a focus on health variables, the results revealed the following facts. In both types of health system, males of working age were characterized by considerably higher rates of treatable mortality from circulatory system diseases, as well as endocrine, nutritional and metabolic diseases. Treatable mortality from circulatory system diseases dominated over mortality from endocrine, nutritional and metabolic diseases. Regarding circulatory system diseases, in countries with a tax-based health system, the results indicated that males annually achieved an average of 49.12 more deaths (per 100,000 males of working age) than females (per 100,000 females of the same age category). In countries with an insurance-based health system, this inequality represented an average of 69.18 more deaths at the expense of males. Based on the mean values, it was also clear that countries with an insurance-based health system reported higher mortality rates than countries which applied a tax-based health system.

### Assessing the Associations Between Health Care Financing, Treatable Mortality, and Economic Prosperity—Bivariate and Multivariate View

This subsection aims to assess the significance of the examined associations between health care financing, treatable mortality in individual diagnosis groups, and the economic prosperity of countries in gender differentiation of the working age population, as well as in the specification of health systems.

The use of specific panel regression models was preceded by testing the assumptions. The balance statistics of panel regression models in the analyzed cases leaned more toward a balanced model. This was supported by the following values of gamma (γ) and nu (ν), and it is true that the closer their value is to 1, the more the panel appears to be balanced. For countries with a tax-based health system, both diagnosis groups acquired γ = 0.8463299 and ν = 0.9060825 when examining all the associations. For countries with an insurance-based health system, these were γ = 0.7470208 and ν = 0.9352060 when examining the associations between health care financing and treatable mortality due to given causes, and γ = 0.7376557 and ν = 0.9380606 when examining the associations between treatable mortality and economic prosperity. The presented coefficients of determination (*R*^2^) provide only an informative value and it is not necessary to consider them in terms of model strength, as the low value is given by a relatively low number of observations due to the classification of the panel data structure, i.e., within diagnosis groups, gender and health system specification.

After regression analysis, a cluster analysis was performed in each diagnosis group in the classification according to gender and health system. In the first place, the data in the individual diagnosis groups were averaged over the analyzed period in each country separately for males and females. The same step was taken in economic indicators, and thus health care financing and economic prosperity (GDP) were first averaged separately in each country for the analyzed period, but gender differentiation was not applied. In the next step, these health and economic outcomes were standardized in the range of 0–1, with a value of 0 indicating the least positive result and a value of 1 indicating the most positive result. In the case of economic indicators, the data were again averaged to create one economic outcome per country. This process created two variables assessing economic indicators and indicators of treatable mortality in individual countries, which were included in the cluster analysis.

### Treatable Mortality From Circulatory System Diseases

This part of the analytical processing is devoted to the evaluation of the investigated associations and their significance in the diagnosis group of circulatory system diseases (CRC). The results of testing the assumptions for the preference of the regression model are shown in Table 2.

**Table 2:** Testing of assumptions for the selection of regression models in the diagnosis group of circulatory system diseases.

		***F* Test–countries**	***F* Test–years**	**Hausman Test**	**Model**
		**(*p*)**	**(*p*)**	**(*p*)**	
**Tax-based health system**					
Males	HF→ CRC	162.988 (<0.001)	0.678 (0.858)	10.655 (0.001)	One-way fixed
	CRC→ GDP	68.552 (<0.001)	5.474 (<0.001)	81.097 (<0.001)	Two-ways fixed
Females	HF→ CRC	98.413 (<0.001)	0.608 (0.915)	12.705 (<0.001)	One-way fixed
	CRC→ GDP	39.985 (<0.001)	5.192 (<0.001)	56.078 (<0.001)	Two-ways fixed
**Insurance-based health system**					
Males	HF→ CRC	196.484 (<0.001)	0.532 (0.961)	2.699 (0.100)	One-way random
	CRC→ GDP	92.320 (<0.001)	2.734 (<0.001)	6.931 (0.008)	Two-ways fixed
Females	HF→ CRC	114.004 (<0.001)	1.082 (0.364)	5.085 (0.024)	One-way fixed
	CRC→ GDP	89.978 (<0.001)	1.948 (0.007)	2.562 (0.109)	Two-ways random

*HF, Health care financing in % of GDP; CRC, Treatable mortality from circulatory system diseases per 100,000 males/females aged 25–64 years; GDP, Gross domestic product per capita*.

From the results in Table 2, it is clear that a one-way fixed effects model and its results were recommended to be taken into account when assessing the significance of most associations between health care financing and treatable mortality due to circulatory system diseases (HF→ CRC). The choice of this model was supported by the results of F tests, which revealed significant effects in the data structure only within the countries that needed to be respected in a one-way variant of the model. At the same time, in these cases, the results of the Hausman test with a *p* < 0.05 testified in favor of a model with fixed effects. An exception was the case in the male population of countries with an insurance-based health system, in which a one-way model was preferred but with random effects, as the result of the Hausman test was not significant. In most cases, a two-ways fixed effects model was recommended to assess the significance of the associations between treatable mortality due to circulatory system diseases and economic prosperity (CRC→ GDP). The results of F tests, where appropriate, showed effects in the data structure in terms of countries and years, and the results of the Hausman test favored a model with fixed effects (*p* < 0.05). An exception could be observed in the female population in countries with an insurance-based health system, in which the preference leaned toward a two-ways model with random effects.

Table 3 presents the results of the evaluation of associations in the diagnosis group of circulatory system diseases, based on which it can be concluded that health care financing appeared to be a significant factor in treatable mortality from circulatory system diseases in males and females aged 25–64 years in both systems. The association with statistical significance at the level of α < 0.001 was confirmed in all analyzed cases in terms of gender and health systems. Simultaneously, negative values for the β coefficient were identified in all cases, indicating that a higher rate of health care financing was associated with a reduction in treatable mortality due to circulatory system diseases and vice versa. When comparing health systems, more pronounced results can be observed in countries with an insurance-based health system, while in a gender comparison, more pronounced results could be observed in the male population.

**Table 3:** Regression analysis—associations between health care financing, treatable mortality from circulatory system diseases and economic prosperity.

			**One-way random effects model**	**One-way fixed effects model**	**Two-ways random effects model**	**Two-ways fixed effects model**
**Tax-based health system**						
Males	HF→ CRC	*R* ^2^	0.544	0.550	0.338	0.044
		α	188.90^***^		145.05^**^	
		β	−12.34^***^	−12.02^***^	−6.88	−2.15^**^
	CRC→ GDP	*R* ^2^	0.592	0.755	0.213	0.009
		α	63668.55^***^		46501.71^***^	
		β	−350.60^***^	−463.12^***^	−163.70^***^	−43.30
Females	HF→ CRC	*R* ^2^	0.469	0.459	0.284	0.006
		α	64.73^***^		49.32^***^	
		β	−4.27^***^	−4.13^***^	−2.36	−0.39
	CRC→ GDP	*R* ^2^	0.518	0.642	0.242	0.001
		α	59125.19^***^		42168.98^***^	
		β	−873.74^***^	−1134.75^***^	−338.81^***^	12.64
**Insurance-based health system**						
Males	HF→ CRC	*R* ^2^	0.202	0.208	0.099	0.027
		α	208.41^***^		196.82^***^	
		β	−13.10^***^	−13.29^***^	−11.55^***^	5.22
	CRC→ GDP	*R* ^2^	0.376	0.392	0.208	0.003
		α	47884.35^***^		31938.93^***^	
		β	−178.95^***^	−187.81^***^	−43.20^***^	−9.40
Females	HF→ CRC	*R* ^2^	0.230	0.241	0.109	0.038
		α	85.60^***^		80.36^***^	
		β	−5.97^***^	−6.19^***^	−5.26^***^	2.43^*^
	CRC→ GDP	*R* ^2^	0.434	0.442	0.289	0.001
		α	46299.33^***^		33180.44^***^	
		β	−442.63^***^	−450.02^***^	−141.75^***^	−5.03

*** *p < 0.001;*

** *p < 0.01;*

* *p < 0.05*.

*The accepted significant results are highlighted in bold*.

With a focus on the associations between treatable mortality from circulatory system diseases and economic prosperity, it is clear that a significance (α < 0.001) was revealed in the female population of countries with an insurance-based health system, when the result of the two-ways random effects model was taken into account. It is also evident that no significant associations were observed in the other cases when considering panel two-ways models with fixed effects. The choice of two-ways variants should be considered correct, but there was room to accept the results of a two-ways model with random effects. This model still appeared to be effective, although it may not be consistent as a model with fixed effects. Therefore, the results of this model could be supported with some caution, which also suggested a limitation of the research. On the other hand, the value of the determination coefficients indicated the possibility to lean toward the two-ways model with random effects. From the results of the two-ways random effects model, it was evident that treatable mortality from circulatory system diseases was negatively associated with economic prosperity. In this way, an increase in GDP was associated with a reduction in treatable mortality from circulatory system diseases and vice versa. These inverse associations with a significance at the level of α < 0.001 were revealed in both health systems and both gender categories of the population.

**Figure 1:**
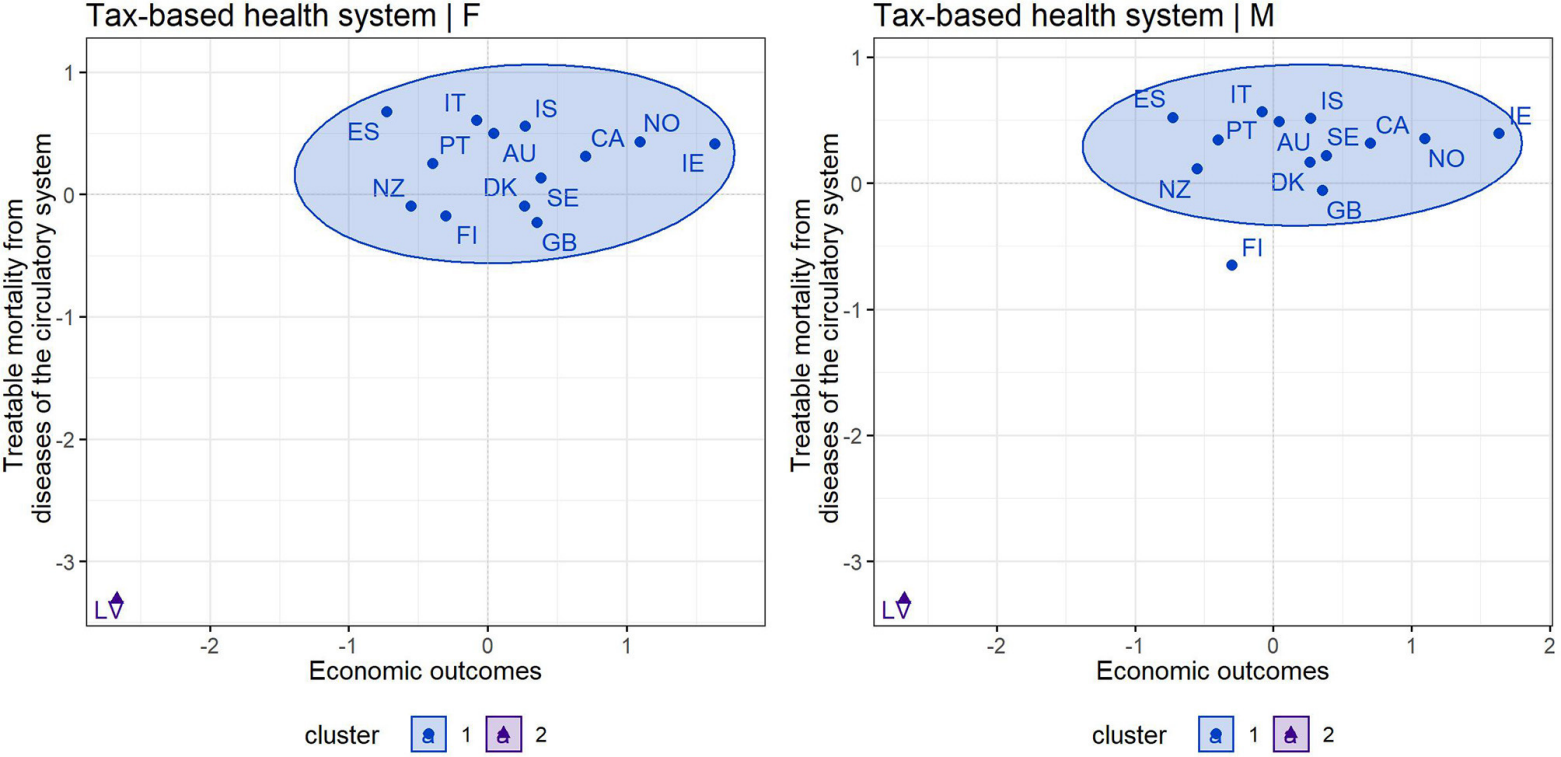
Cluster map—economic outcomes and treatable mortality from circulatory system diseases for countries applying a tax-based health system—females (F) and males (M).

**Figure 2:**
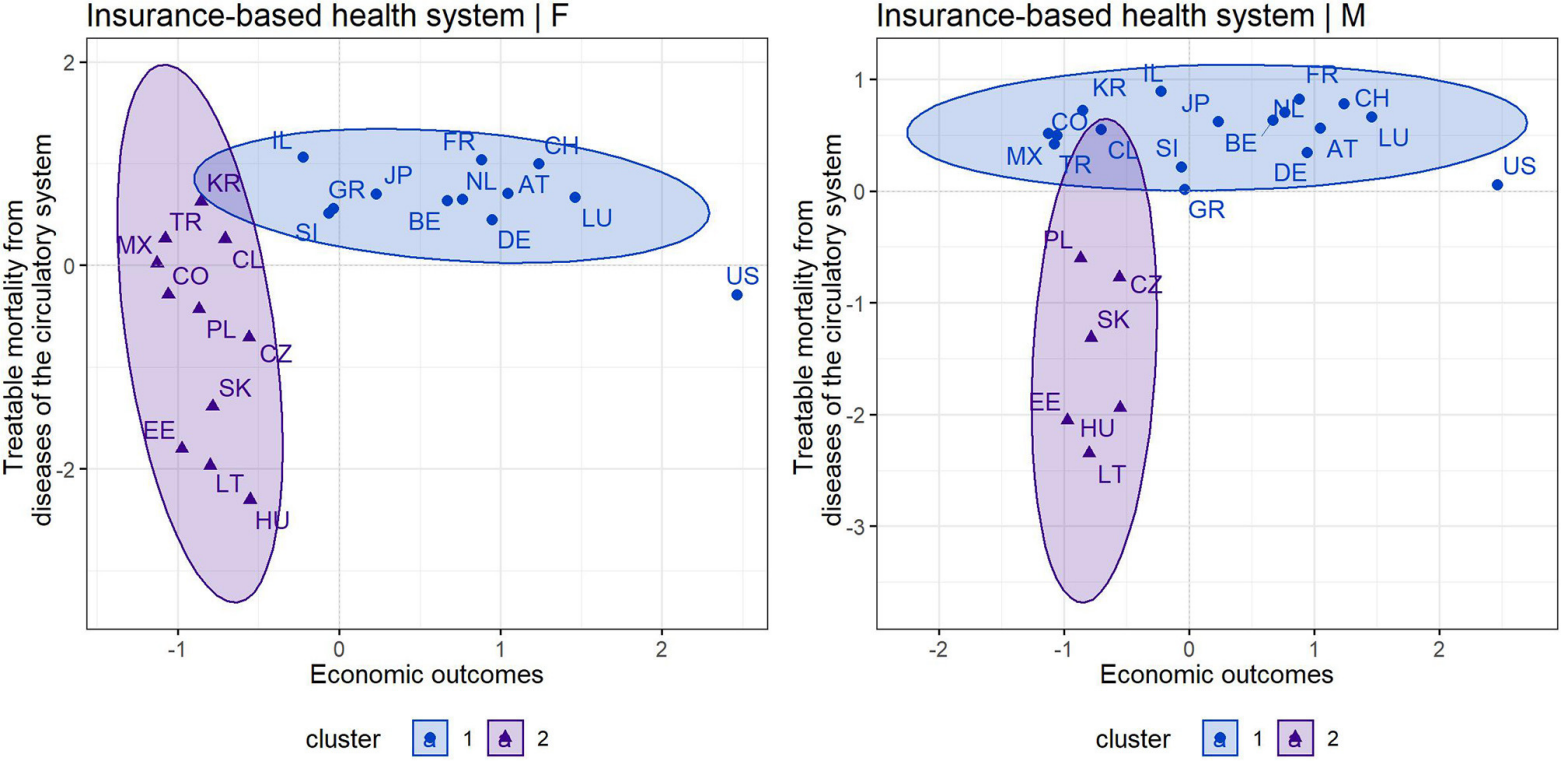
Cluster map—economic outcomes and treatable mortality from circulatory system diseases for countries applying an insurance-based health system—females (F) and males (M).

The cluster maps shown in Figures 1, 2 provided a grouping of countries based on an assessment of treatable mortality from circulatory system diseases and economic outcomes (a variable combining health care financing and the economic prosperity of countries). Figures 1, 2 also show that the silhouette method recommended two clusters to form organized groups in both health systems and in both gender categories. With a focus on countries with a tax-based health system (Figure 1), cluster 1, which could be considered the most positive in terms of assessment, included all countries except Latvia. This country acquired the least positive assessment of economic and health outcomes in the male and female population, indicating an undesirable position in the lower left margin. Regarding countries with an insurance-based health system (Figure 2), the countries in cluster 1 could be considered as the countries with the most positive outcomes. These countries were characterized by lower treatable mortality and higher economic conditions. The positive outcomes were dominated by countries such as Switzerland, Luxembourg and France. On the other hand, countries such as Lithuania, Hungary, Estonia, and Slovakia in cluster 2 could be considered the least positive. These countries reported higher treatable mortality and lower economic outcomes.

### Treatable Mortality From Endocrine, Nutritional, and Metabolic Diseases

The evaluation of individual associations and their significance in the diagnosis group of endocrine, nutritional and metabolic diseases (END) is presented in this part of the analytical process. Also in this diagnosis group, a panel diagnostic for model selection was first performed and its results are presented in Table 4.

**Table 4:** Testing of assumptions for the selection of regression models in the diagnosis group of endocrine, nutritional and metabolic diseases.

		***F* Test–countries**	***F* Test–years**	**Hausman Test**	**Model**
		**(*p*)**	**(*p*)**	**(*p*)**	
**Tax-based health system**					
Males	HF→ END	126.372 (<0.001)	0.372 (0.996)	1.638 (0.201)	One-way random
	END→ GDP	9.627 (<0.001)	5.995 (<0.001)	0.459 (0.498)	Two-ways random
Females	HF→ END	92.657 (<0.001)	0.527 (0.961)	4.054 (0.044)	One-way fixed
	END→ GDP	10.486 (<0.001)	5.705 (<0.001)	11.149 (0.001)	Two-ways fixed
**Insurance-based health system**					
Males	HF→ END	416.679 (<0.001)	0.198 (1.000)	0.073 (0.786)	One-way random
	END→ GDP	61.336 (<0.001)	4.260 (<0.001)	0.016 (0.901)	Two-ways random
Females	HF→ END	1240.726 (<0.001)	0.194 (1.000)	0.011 (0.916)	One-way random
	END→ GDP	74.341 (<0.001)	4.073 (<0.001)	5.949 (0.015)	Two-ways fixed

*HF, Health care financing in % of GDP; END, Treatable mortality from endocrine, nutritional and metabolic diseases per 100,000 males/females aged 25–64 years; GDP, Gross domestic product per capita*.

Based on the results in Table 4, it is possible to state that a one-way random effects model should be preferred to assess the significance of most associations between health care financing and treatable mortality due to endocrine, nutritional and metabolic diseases (HF→ END). Specifically, this model was recommended in the case of the male population in countries with a tax-based health system, as well as in the case of both gender categories in countries with an insurance-based health system. The preference for this variant of the panel regression model was based on the results of *F* tests indicating significant effects in the data structure only within countries and on the results of the Hausman test with a *p* > 0.05 recommending a model with random effects. Conversely, a one-way model with fixed effects was preferred in the case of the female population of countries with a tax-based health system, as the results of the Hausman test showed a *p* < 0.05. In terms of the associations between treatable mortality due to endocrine, nutritional and metabolic diseases, and economic prosperity (END→ GDP), a two-ways variant of the model was preferred, as the results of *F* tests revealed significant effects in the data structure across countries and years. The results of the Hausman test with a *p* > 0.05 supported the choice of a model with random effects in the male population in both health systems, and the results with a *p* < 0.05 were in favor of a model with fixed effects in the female population in both health systems. The main results of the panel regression models are shown in Table 5.

**Table 5:** Regression analysis—associations between health care financing, treatable mortality from endocrine, nutritional and metabolic diseases and economic prosperity.

			**One-way random effects model**	**One-way fixed effects model**	**Two-ways random effects model**	**Two-ways fixed effects model**
**Tax-based health system**						
Males	HF→ END	*R* ^2^	0.039	0.020	0.002	0.003
		α	8.27^***^		7.74^***^	
		β	−0.18^*^	−0.17 ×	−0.12	−0.09
	END→ GDP	*R* ^2^	0.045	0.023	0.063	0.024
		α	42293.70^***^		34656.26^***^	
		β	−1103.40^**^	−1098.04	−447.19^*^	−432.86
Females	HF→ END	*R* ^2^	0.146	0.127	0.021	0.001
		α	6.00^***^		5.35^***^	
		β	−0.28^***^	−0.28**^*^	−0.21 ×	0.02
	END→ GDP	*R* ^2^	0.167	0.160	0.166	0.019
		α	7942.43^***^		32271.36^***^	
		β	−3704.73^***^	−4379.47^***^	−117.62	573.28
**Insurance-based health system**						
Males	HF→ END	*R* ^2^	0.011	0.010	0.039	0.015
		α	12.83^***^		12.83^***^	
		β	−0.29^*^	−0.28	−0.29	−0.53
	END→ GDP	*R* ^2^	0.017	0.008	0.072	0.022
		α	31839.40^***^		29072.91^***^	
		β	−306.32^*^	−281.49	−215.14**^*^	−203.86
Females	HF→ END	*R* ^2^	0.106	0.109	0.049	0.001
		α	11.11^***^		11.11^***^	
		β	−0.52**^*^	−0.51^*^	−0.52^*^	−0.02
	END→ GDP	*R* ^2^	0.106	0.169	0.095	0.010
		α	39280.76^***^		29254.50^***^	
		β	−1499.83^***^	−2348.16^*^	−337.15**^*^	−265.73

*** *p < 0.001;*

** *p < 0.01;*

* *p < 0.05; × p < 0.1*.

*The accepted significant results are highlighted in bold*.

The results in Table 5 point to the fact that even in the diagnosis group of endocrine, nutritional and metabolic diseases, health care financing played an important role in both health systems. Proof of this is the statistical significance of associations in both male and female populations. It can also be noted that in both health systems, a greater strength of associations with significance at the level of α < 0.001 was identified in the female population. In terms of the male population, associations with less strength and statistical significance at the level of α < 0.05 were found in both health systems. Based on the negative β coefficients, an increase in health care financing was associated with a reduction in treatable mortality in this diagnosis group. When comparing the systems, it can be seen that the financing of health care was a more significant aspect in countries with an insurance-based health system.

Focusing on the associations between treatable mortality from endocrine, nutritional and metabolic diseases and economic prosperity, a significant negative association was found in the male population in both health systems. For this diagnosis group, a reduction in the treatable mortality in males of working age was associated with an increase in GDP. A different situation could be observed in the female population in both health systems, as no significant association was confirmed by the preferred two-ways model with fixed effects. As in the previous diagnosis group, there was a room for cautious acceptance of the results of the two-ways random effects model, which revealed a significant negative association in the female population of countries with an insurance-based model. This could be considered a limitation.

**Figure 3:**
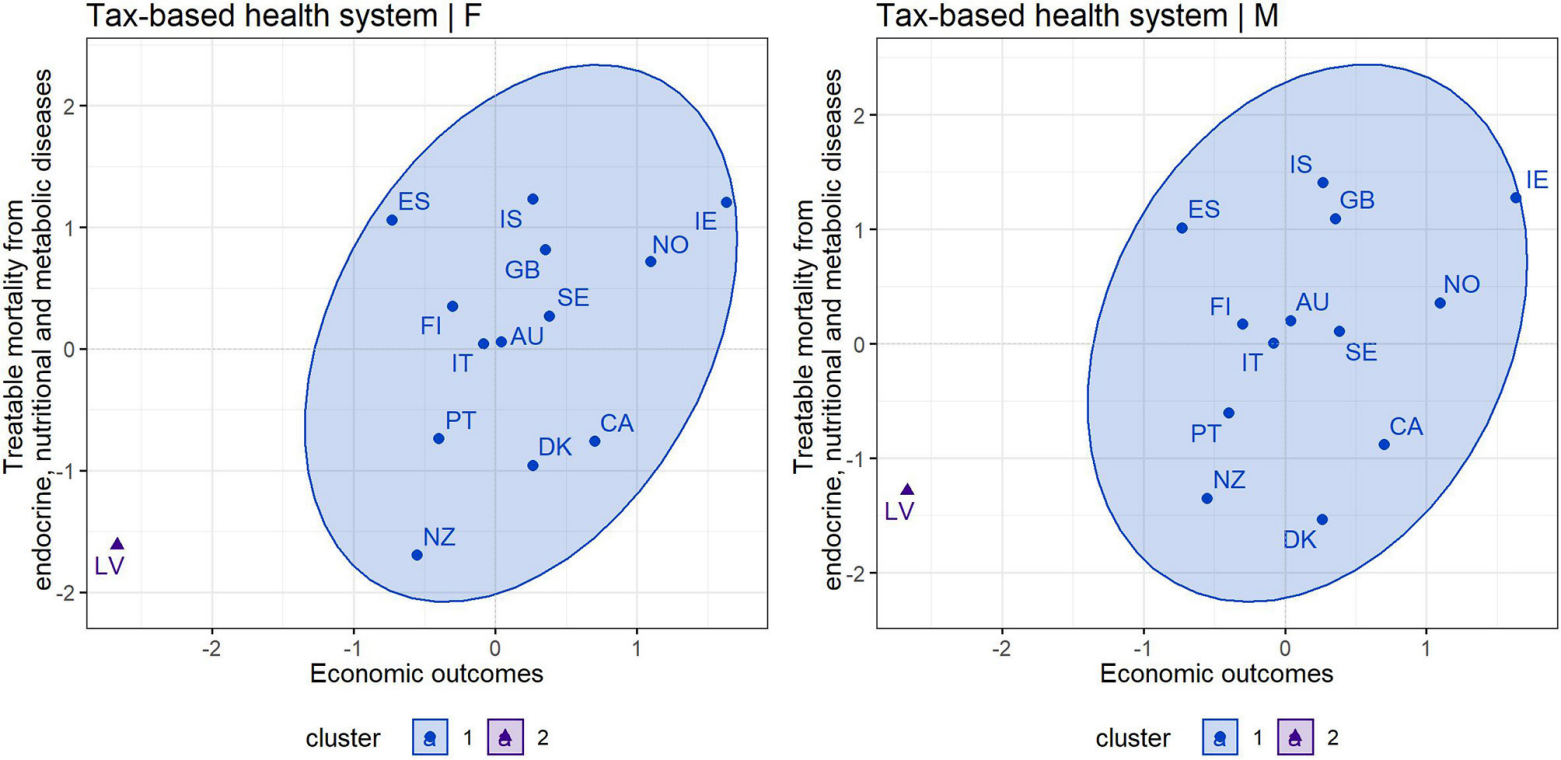
Cluster map—economic outcomes and treatable mortality from endocrine, nutritional and metabolic diseases for countries applying a tax-based health system—females (F) and males (M).

**Figure 4:**
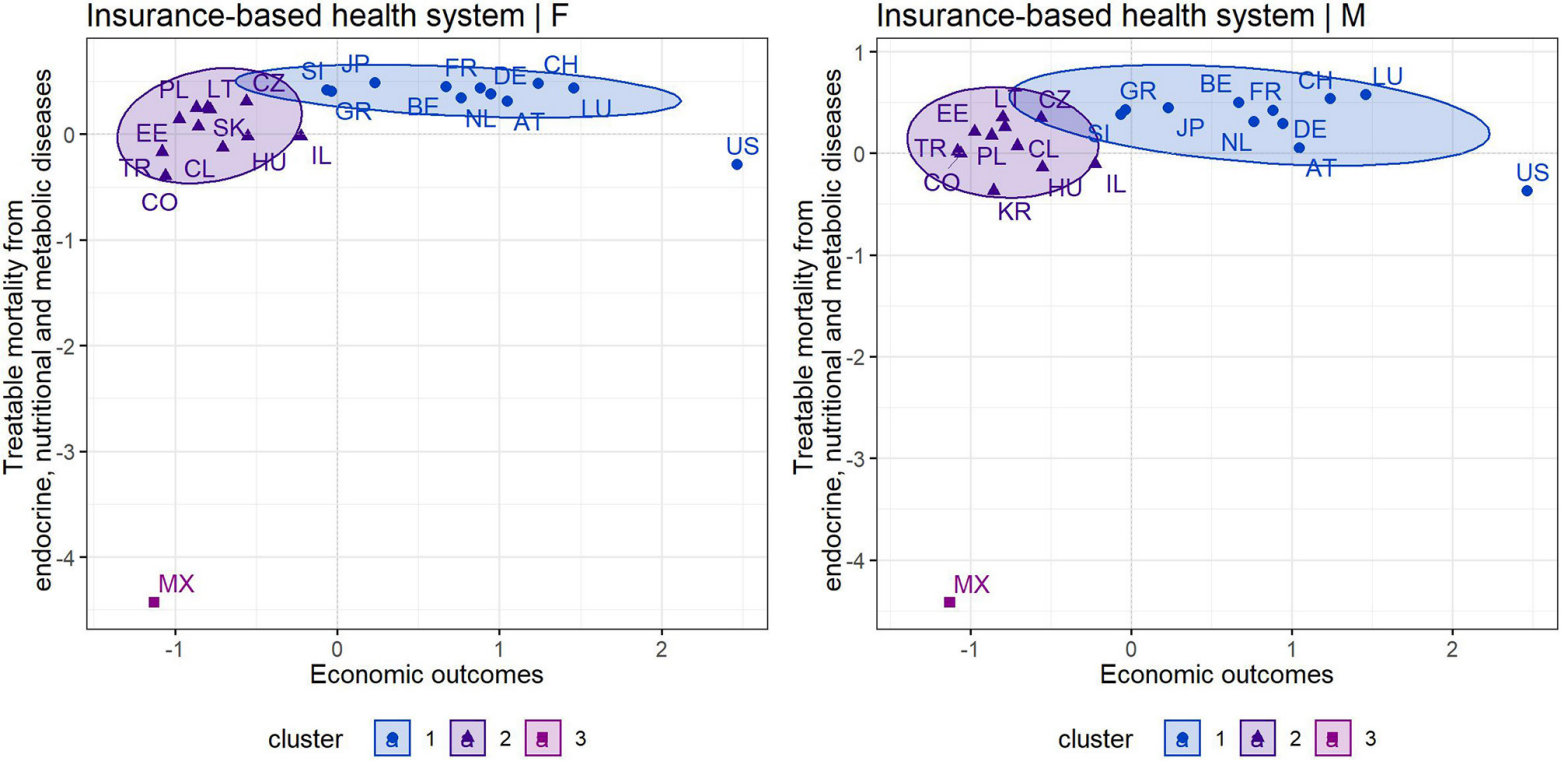
Cluster map—economic outcomes and treatable mortality from endocrine, nutritional and metabolic diseases for countries applying an insurance-based health system—females (F) and males (M).

As in the previous diagnosis group, Figures 3, 4 show the cluster maps that provide grouping of countries based on an assessment of treatable mortality from endocrine, nutritional and metabolic diseases and economic outcomes (a variable combining health care financing and economic prosperity of countries). The silhouette method recommended two clusters to form organized groups of countries with a tax-based health system in both gender categories, while three clusters were recommended for countries with an insurance-based system and for both gender categories. In countries with a tax-based health system (Figure 3), a similar situation could be observed as in the previous diagnosis group. Thus, cluster 1 included all countries except Latvia with the least positive outcomes. However, the countries in cluster 1 were not as homogeneous as in the previous diagnosis group. At this point, it is possible to pay increased attention to countries such as Denmark, New Zealand, Canada and Portugal, which acquired less positive assessment of health outcomes compared with other countries in this cluster, i.e., higher treatable mortality from endocrine and metabolic diseases. Focusing on countries with an insurance-based health system (Figure 4), Mexico acquired the least positive assessment of economic and health outcomes in the male and female population, indicating an undesirable position in the lower left margin. This country can be considered as a remote country among other countries due to its high level of treatable mortality recorded in this diagnosis group. Consequently, the countries with less positive outcomes included Colombia and Turkey. Attention can also be drawn to the United States, which showed high economic outcomes, but despite its level, the country recorded a high treatable mortality rate.

## Discussion

### Insight Into Treatable Mortality in OECD Countries

By comparing the examined diagnosis groups, the presented study and its results showed that diseases of the circulatory system contributed to a greater extent to treatable mortality in OECD countries with a tax-based health system and an insurance-based system. These findings are consistent with the results of Jarčuška et al. (41), as well as the results of the WHO survey (42) on the leading causes of deaths from a global perspective. The survey showed that ischemic heart diseases and strokes, belonging to the diagnosis group of circulatory system diseases, occupy the first ranks in a negative sense. At the same time, Dagenais et al. (43) confirmed that cardiovascular mortality is the largest health burden in low-, middle- and high-income countries. A positive signal is the declining trend in treatable mortality (44–46), while Weber and Clerc (47) attributed this trend mainly to a reduction in mortality from diseases of the circulatory system.

In terms of gender, the results clearly showed that males were characterized by a higher average mortality than females in the examined diagnosis groups of treatable mortality. These findings are consistent with the findings of other studies that confirmed a higher mortality rate for males, reflecting the fact that females live longer (48–50). In this regard, Le et al. (51) revealed that it is the higher male mortality from diseases of the circulatory and respiratory systems that appears to be the main contributing factor to this gender gap. Gender differences in avoidable mortality were also revealed by Westerling (52) and Sundmacher (53), while the results clearly showed that males are higher risk of deaths due to treatable and preventable diseases than females (54, 55). This study on treatable mortality is consistent with previous findings. The problem should take into account biological differences between males and females, while genetic factors, immune system responses, hormonal activity or the course of diseases that can lead to such differences (48). It is also important to realize that males have different patterns of health behavior, which are manifested in a reluctance to seek medical treatment, a reluctance to adhere to treatment, and a lack of reporting of their health problems (48). All of these factors can be critical to treatable deaths and should be taken into account in health care provision and strategy development. This can be an important impetus for leaders of health systems, which strive to improve the health of the population, reduce inequalities and ensure quality health care. According to the results, monitoring the health status of males appears to be justified.

In addition to gender differences, cluster maps also indicated differences between individual OECD countries. Similar findings were revealed by Weber and Clerc (47), who pointed to large differences in treatable mortality between European countries at the international level, but also at their regional level. Focusing on the individual indicators of treatable mortality in both examined systems, it can be concluded that countries applying an insurance-based health system were characterized by less positive health outcomes than countries applying a tax-based health system. These findings differ from those of van der Zee and Kroneman (16), who considered an insurance-based health system to be a more positive system. On the other hand, these authors took into account overall mortality, life expectancy, and patient satisfaction in their research. In the presented study, countries with an insurance-based health system were characterized by a higher rate of treatable mortality in both diagnosis groups. The results of a descriptive analysis of health care financing pointed to the fact that countries applying an insurance-based health system spend less on health care than countries applying a tax-based health system. Already at this point, it was possible to see a certain link between health care financing and treatable mortality.

### The Role of Health Care Financing in Treatable Mortality

In both diagnosis groups, health care financing has been shown to play an important role in the treatable mortality of both males and females in both systems. The regression analysis revealed a significant negative association between health care financing and treatable mortality in each analyzed case, with greater strength of the associations observed in countries with an insurance-based health system. Also, a greater strength of the significant association was identified for diagnosis group of circulatory system diseases. These findings indicated that an increase in health expenditure may be associated with a reduction in treatable mortality of females and males of working age in both diagnosis groups. The results of the regression analysis also indicated that the associations were greater in the male population than in the female population. Despite the findings of several authors suggesting that health care financing is not such an important aspect in population health (13, 14), the findings of this study support the idea that a level of health expenditure is associated with treatable mortality in each of the examined health systems. Under ceteris paribus conditions, a higher level of health expenditure may be reflected in reduced treatable mortality. These findings are consistent with the findings of studies examining commonly used health indicators that involve more or less the strength of other factors (7–9). These were overall mortality, infant mortality, morbidity of the population or life expectancy, which also include social and economic factors affecting the health of the population (3, 10, 11). In terms of overall mortality, many studies confirmed the significant impact of health expenditure (56, 57), but treatable mortality is a specific health indicator separated from overall mortality that is able to capture the contribution of health care. The findings in this study are consistent with the findings of Heijink et al. (23) or Kjellstrand et al. (58), who provided evidence that countries with a higher level of health care expenditure report lower avoidable mortality.

### The Role of Treatable Mortality in Economic Prosperity

It should be borne in mind that treatable mortality includes avoidable deaths that are considered unnecessary and dependent on health care interventions and secondary prevention (21). For this reason, there is an assumption that, in the absence of these unnecessary deaths, people would continue to contribute to the economic productivity of countries through their activity. In this context, treatable mortality can represent a significant economic burden. This assumption was also based on a study conducted by Alkire et al. (24), whose findings revealed that the unfoundedness of treatable mortality brings economic losses reflected in a decline in countries' GDP. Based on the regression analysis performed in this study, it can be concluded that a reduction of treatable mortality due to circulatory system diseases and endocrine and metabolic diseases in people of working age is associated with an increase in economic prosperity. At this point, it can be emphasized that these are diagnosis groups that include diseases of civilization, which is a great challenge in many countries, and these findings underline the importance of treating individual diseases. In the diagnosis group of endocrine and metabolic diseases, it has been shown that reducing mortality, especially in males of working age, could be economically beneficial in countries applying a tax-based health system as well as an insurance-based health system. It was also possible to speak cautiously of a significant association in the female population in countries with an insurance-based health system. Regarding diseases of the circulatory system with the greatest health and economic burden (42, 43), the results supported the assumption that reducing mortality in this diagnosis group is associated with increasing economic prosperity of countries. All these findings support the idea that the financing of health care can be reflected in the economic prosperity of countries through the variability of population health. The presented findings are in line with evidence that reducing population mortality improves economic development and prosperity (17–19, 24). Thus, in order to achieve economic benefits, emphasis needs to be placed on reducing mortality and, among other interventions, increased health care financing can help.

### Assessment of Individual Countries

The cluster analysis offered the categorization of countries into clusters according to the assessment of treatable mortality outcomes and economic outcomes. In countries with a tax-based health system, almost all countries formed a homogeneous cluster in terms of assessing economic outcomes and treatable mortality from circulatory system diseases. The only exception was Latvia with the least positive outcomes among these countries. In countries with an insurance-based health system, higher treatable mortality from circulatory system diseases and lower economic outcomes were found in Lithuania, Hungary, Estonia, and Slovakia. On the contrary, Switzerland, Luxembourg and France could be considered the most positive countries with an insurance-based health system. With a focus on the endocrine and metabolic treatable causes of deaths, Latvia, as well as Denmark, New Zealand, Canada, and Portugal with a tax-based health system have taken a position indicating higher mortality. Mexico showed the least positive assessment of treatable mortality from endocrine and metabolic diseases and economic outcomes compared to other countries with an insurance-based health system. In addition to this country, Colombia and Turkey also reported less positive outcomes. Special attention should also be paid to the United States, which, despite high levels of health expenditure and GDP, has not acquired such positive results in terms of treatable mortality as other developed Western countries. This could be due to the high cost of health care in this country. In this comparative manner, the findings in this study are consistent with the findings of authors such as Pritchard et al. (59), who examined this issue in terms of specific mortality, but without taking into account the treatability of diseases. Regarding avoidable mortality, the findings agree with those of Kjellstrand et al. (58). The authors of both studies considered the health system of the United States to be the least efficient (58, 59). With a high level of health expenditure, more positive health outcomes could be expected. The serious situation in the United States is also underlined by the fact that some countries reported lower expenditure but achieved more positive health outcomes. All these findings confirm the fact that health is a source of comparative economic development in countries (2).

At this point, it is possible to highlight the results of the regression analysis, which revealed that the financing of health care represents one of the important tools for improving the health of the population. The findings indicated that if less positively evaluated countries in the cluster analysis increase their level of financing for health care, this could translate into lower treatable mortality and consequently higher economic prosperity. This could improve their position, and therefore it is possible to look at less positively evaluated countries as countries with great potential and opportunities for improvement. At the same time, other countries with a positive assessment can be an inspiration for these countries.

### Policy Implications

A look at the problem in this study suggests that health expenditure is one of the important factors in the treatable mortality of both males and females, which supports the need to take this factor into account when developing strategic plans. The financing of health care is highlighted as key aspect in universal health coverage (24). Moreover, health care financing can be reflected in economic prosperity through health variability in the working age population, as treatable mortality is considered an economic burden. Although reducing treatable mortality is a great motivation for public health leaders to increase health care financing, the importance for economic prosperity may be a more compelling argument for government representatives. The considerations and recommendations from this study can be applied both in a tax-based health system and in an insurance-based health system, as each system should be able to be more efficient.

As there were clear differences in treatable mortality across OECD countries (41, 44), policy makers should also address the issue of closing this health gap. Differences in treatable mortality were also evident between population groups within one country, and in order to achieve comparable outcomes with other countries, an internal problem needs to be addressed first (60). It is very difficult to get closer to successful countries if there are obvious health disparities between geographical regions and provinces (61, 62). Each country is specific and this fact should be taken into account when formulating health policies. For instance, poverty is closely linked to treatable mortality (63, 64) and in this context it should be borne in mind that if the health system provides health care to populations with higher poverty and other socio-economic specificities, it may be reflected also in a greater need for financial resources in the system (20). Treatable mortality should be interpreted both as an indicator of health care quality and as a reflection of the unequal distribution of socio-economic resources (65). Both health systems examined in this study should increase efforts to enhance treatable survival. In order to increase the effectiveness of health systems, special emphasis should be placed on the development of rules, standards and regulations with a view to a more integrated and equitable implementation of health policies and financing (66). These efforts can also be economically beneficial, as the rate of associations between treatable mortality and economic prosperity has not been negligible, which has also been shown in the research of Alkire et al. (24). Thus, when creating public policies, it is appropriate to consider all the facts resulting from the presented study. However, it should be emphasized that financing is not the only important factor and other country-specific factors should be taken into account.

In view of the above-mentioned facts, international cooperation is needed to address global challenges in terms of treatable mortality and related economic risks. This cooperation would strengthen the global health system by improving collaboration and coordination across international organizations. This effort can fill gaps in knowledge with respect to treatable causes of death, research and development needs, financing models, and the social and economic impacts of potential threats. Also, this international response would provide high-level, evidence-based recommendations for managing the global risks associated with treatable mortality (67).

### Strengths and Limitations

The study clarified the economic perspective of treatable mortality and its strength lies in a multi-level investigation. Thus, the study explained the associations between health care financing and treatable mortality, and, subsequently, the associations between treatable mortality and economic prosperity in a comprehensive data classification, which made it possible to avoid superficial findings. The analyzes respected specific diagnosis groups, gender differentiation and individual health systems and their results provided a deeper insight to the issue. Based on multidimensional research and multilevel classification, it was possible to answer the question of what strength of associations exists in individual health systems, individual diagnosis groups, and gender categories. The study also provided a comparison of countries and contributed to the creation of a valuable platform of evidence at national and international level. The results of this study were able to clarify the comprehensive view of the associations that existed in the scientific community. Last but not least, given the low availability of treatable mortality data in specific diagnosis groups in individual countries, it should be emphasized that the study contains the most up-to-date data on treatable mortality for up to 37 OECD countries.

This research did not avoid the limitations that could be addressed in future research. In this sense, it should be pointed out that the findings concern only OECD countries and cannot be generalized to less developed countries or countries with other health systems. The unbalanced structure of the panel data is also a weakness of the research, but it should be noted that not every country reported the number of specific treatable deaths each year and the presented research covered the widest possible range of treatable mortality in 37 OECD countries. Limitations also include the fact that the results of a regression model other than the recommended one were taken into account. Nevertheless, a reliable model was chosen. Regarding the limitations of the models, it should be noted that the models used in this study did not examine causality as such, and therefore the results cannot be interpreted as causal relationships. All results can only be seen in terms of associations, while a consideration of causal relationships can be misleading. Last but not least, it must be emphasized that the financing of health care is not the only factor in the health of the population. Thus, the results should not be considered the only right pathway. Factors such as doctors' qualifications, their working conditions, equipment, access to medicines, or hospital management play an important role in treatable mortality. Future research should address them.

## Conclusion

This study focuses on the associations between health care financing, specific treatable mortality of males and females of working age, and economic prosperity in the classification of health systems applied in OECD countries. The main aim was met by descriptive analysis, panel regression analysis, and cluster analysis. Based on the results, it was possible to answer the research questions in the affirmative. The treatable mortality was represented by two diagnosis groups, namely circulatory system diseases and endocrine, nutritional, and metabolic diseases. In addition to these diagnosis groups, the analyzes respected the specification of gender and health systems, which provided a deeper insight into the main findings. There were significant negative associations between health care financing and treatable mortality, as well as between treatable mortality and economic prosperity in both health systems. The study also provides an international comparison of OECD countries, on the basis of which less positive and more positive countries were identified. The main findings of the study supported the idea that the economic life of countries is linked to the health of the population. Healthy individuals are considered the driving force of the economy, but sick individuals represent a potential burden in times of disease and death. In any case, it should be borne in mind that investing in public health can translate into economic benefits, and therefore the financing of health care requires special attention. This is true, although it is not the only significant factor. Successful management of health systems requires an individualized approach to decision-making that takes into account proven evidence and implements it in procedures aimed at achieving health goals. This study would help policy makers to understand the nature of the problem from the presented perspective and to make the right decisions given the diversity of systems, population groups and other country specificities. The study supports the strengthening of health systems and offers a basis for further research and the creation of international databases of evidence.

## Data Availability Statement

Publicly available datasets were analyzed in this study. Data on health expenditure can be found in the OECD database: https://data.oecd.org/healthres/health-spending.htm#indicator-chart. Data on GDP can be found in the OECD database: https://data.oecd.org/gdp/gross-domestic-product-gdp.htm. Data on treatable mortality can be found in the WHO database: https://www.who.int/data/data-collection-tools/who-mortality-database?fbclid=IwAR2gVBBqbMEUf6Y1g505FjN_hg77TkINf_VWGO3-efYrTr-J9sC7_Wkpy7Q. Data on population can be found in the United Nations database: https://population.un.org/wup/.

## Ethics Statement

Ethical approval was not required for the study on human participants in accordance with the local legislation and institutional requirements. Written informed consent for participation was not required for this study in accordance with the national legislation and the institutional requirements.

## Author Contributions

VI, BG, GS, and SK: conceptualization, investigation, visualization, and writing—review and editing. BG and SK: original draft preparation, supervision, project administration, and funding acquisition. VI: methodology, formal analysis, data curation, and original draft preparation. BG: resources. All authors contributed to manuscript revision, read, and approved the submitted version.

## Funding

This research was funded by the Scientific Grant Agency of the Ministry of Education, Science, Research, and Sport of the Slovak Republic and the Slovak Academy Sciences and Quantification of Environmental Burden Impacts of the Slovak Regions on Health, Social and Economic System of the Slovak Republic as part of the research project VEGA 1/0797/20.

## Conflict of Interest

The authors declare that the research was conducted in the absence of any commercial or financial relationships that could be construed as a potential conflict of interest.

## Publisher's Note

All claims expressed in this article are solely those of the authors and do not necessarily represent those of their affiliated organizations, or those of the publisher, the editors and the reviewers. Any product that may be evaluated in this article, or claim that may be made by its manufacturer, is not guaranteed or endorsed by the publisher.
